# Effect of a Mobile Phone–Based Glucose-Monitoring and Feedback System for Type 2 Diabetes Management in Multiple Primary Care Clinic Settings: Cluster Randomized Controlled Trial

**DOI:** 10.2196/16266

**Published:** 2020-02-26

**Authors:** Yeoree Yang, Eun Young Lee, Hun-Sung Kim, Seung-Hwan Lee, Kun-Ho Yoon, Jae-Hyoung Cho

**Affiliations:** 1 Division of Endocrinology and Metabolism, Department of Internal Medicine, Seoul St Mary’s Hospital College of Medicine The Catholic University of Korea Seoul Republic of Korea; 2 Catholic Smart Health Care Center The Catholic University of Korea Seoul Republic of Korea; 3 Department of Medical Informatics College of Medicine The Catholic University of Korea Seoul Republic of Korea

**Keywords:** diabetes mellitus, type 2, primary care, mHealth, telehealth

## Abstract

**Background:**

Recent evidence of the effectiveness of mobile phone–based diabetes management systems is generally based on studies conducted in tertiary hospitals or professional diabetes clinics.

**Objective:**

This study aimed to evaluate the clinical efficacy and applicability of a mobile phone–based glucose-monitoring and feedback system for the management of type 2 diabetes mellitus (T2DM) in multiple primary care clinic settings.

**Methods:**

In this multicenter, cluster-randomized controlled, open trial, 13 primary care clinics in Seoul and other large cities in South Korea were voluntarily recruited. Overall, 150 (9 clinics) and 97 (4 clinics) participants with T2DM were assigned to the intervention and control groups, respectively (2:1 allocation). Every month, participants in both groups attended face-to-face physicians’ consultation for the management of diabetes in the clinic. For the intervention group, participants were required to upload their daily self-monitoring of blood glucose (SMBG) results using the mobile phone app in addition to outpatient care for 3 months. The results were automatically transmitted to the main server. Physicians had to check their patients’ SMBG results through an administrator’s website and send a short feedback message at least once a week. At baseline and 3 months, both groups had anthropometry and blood tests, including hemoglobin A_1c_ (HbA_1c_), and responded to questionnaires about treatment satisfaction and compliance.

**Results:**

At 3 months, participants in the intervention group showed significantly more improvement in HbA_1c_ (adjusted mean difference to control −0.30%, 95% CI −0.50 to −0.11; *P*=.003) and fasting plasma glucose (−17.29 mg/dL, 95% CI −29.33 to −5.26; *P*=.005) than those in the control group. In addition, there was significantly more reduction in blood pressure, and the score regarding treatment satisfaction and motivation for medication adherence increased more in the intervention group than in the control group. In the subgroup analyses, the effect on glycemic control was more significant among younger patients and higher baseline HbA_1c_ levels.

**Conclusions:**

The mobile phone–based glucose-monitoring and feedback system was effective in glycemic control when applied in primary care clinic settings. This system could be utilized effectively with diverse institutions and patients.

**Trial Registration:**

Clinical Research Information Service (CRIS) https://tinyurl.com/tgqawbz

## Introduction

### Background

Type 2 diabetes mellitus (T2DM) is a worldwide epidemic that is a major socioeconomic burden on the global health care system [1]. Poorly managed T2DM is associated with increased risks of micro and macrovascular complications and premature mortality. Therefore, it is important to steadily achieve the target levels of multiple risk factors, including hyperglycemia, hypertension, dyslipidemia, and obesity, to prevent diabetic morbidities and mortality [2]. Although diabetes is a lifestyle-related disease that requires daily self-management [3], the current diabetes management system largely depends on intermittent short face-to-face interviews at outpatient clinics and medication prescriptions. Consequently, it is not easy for patients to maintain constant care for diabetes in their daily lives.

There have been many attempts to introduce self-management support systems for patients with T2DM on the basis of information technologies (ITs), including Web-based interventions [4-7]. Since the introduction of the smartphone in 2007, mobile phone app-based monitoring systems have taken a major position as intervention modalities, and evidence of their effectiveness in diabetes management has also been accumulated in different types of groups [8-11]. However, previous positive results of mobile phone use in diabetes management were mostly reported by studies involving patients in tertiary hospitals or professional diabetes clinics [12,13]. A large number of patients with T2DM receive treatment at primary care clinics. Physicians at these clinics are not always experts on diabetes; furthermore, most of them have no experience with IT-based diabetes management systems. Implementation of IT-based systems into the primary care service is necessary and inevitable for sharing appropriate diabetes management systems and, ultimately, reducing diabetes-related complications [12]. Therefore, it is necessary to verify the effectiveness of mobile phone–based monitoring systems in primary care clinic settings, where there is a relative lack of specialized workforce and diabetes education environments compared with large hospitals.

### Objectives

This study aimed to evaluate the clinical efficacy and applicability of an interactive, mobile phone–based monitoring and feedback system for T2DM management in primary care clinic settings by assessing its effect on glycemic control and other combined metabolic risk factors such as hypertension, dyslipidemia, and obesity. We also evaluated participants’ treatment satisfaction and their motivation and knowledge related to long-term medication adherence in chronic diseases.

## Methods

### Study Design and Participants

We performed a 3-month, multicenter, cluster-randomized, open trial. Eligibility criteria for primary care clinics were being located in Seoul and other major cities in South Korea, having a patient pool with T2DM, and access to internet services at the clinic. At first, 17 clinics were voluntarily recruited after open research briefing. Among them, 4 clinics declined to participate, and 13 clinics were randomized (Multimedia Appendix 1). Clinics were the unit of randomization and intervention. A research statistician not involved with this study generated the random allocation sequence using SAS software, version 9.3 (SAS Institute Inc, Cary, the United States). Each clinic was sequentially allocated to an intervention or control group after registration with no masking. Finally, 9 and 4 (2:1 allocation) of the 13 primary care clinics were assigned to the intervention and control groups, respectively.

A notice about study methods and participant recruitment was posted on the bulletin board at each clinic. Subjects with T2DM who agreed to participate voluntarily in this clinical study were screened. Eligible subjects were over 18 years of age, had T2DM for at least one year, could use mobile phones or internet services at home, and had baseline hemoglobin A_1c_ (HbA_1c_) levels between 7% (53 mmol/mol) and 10% (86 mmol/mol). We excluded subjects with type 1 diabetes and insulin pump users, subjects with any significant medical disease (such as active cancer, recent stroke, or myocardial infarction), subjects with severe diabetic complications (moderate to severe nonproliferative diabetic retinopathy, proliferative diabetic retinopathy, serum creatinine >1.5 or 1.4 mg/dL for men or women, respectively, or aspartate aminotransferase [AST] or alanine aminotransferase [ALT] levels >3× the upper normal limit), and subjects who had not been taking stable doses of diabetes medications during the 3 months before enrollment. Subjects of other clinical trials or plans could not participate in this study either.

This study was conducted according to the principles expressed in the Declaration of Helsinki. The protocol was reviewed and approved by the Public Institutional Bioethics Committee designated by the Ministry of Health and Welfare (P01-201504-11-002). Written informed consent was obtained from all participants. The study was registered with the Clinical Research Information Service (CRIS number: KCT 0002554).

### Intervention

After screening, participants who met all inclusion criteria at the intervention clinic were registered on the medical staff website and downloaded a mobile phone app (Hicare smart K, Insung information). Then, the physicians of the primary care clinic educated participants on the individual management targets (glycemic, blood pressure [BP], lipid profile, and body weight) on the basis of the medical guidelines of the Korean Diabetes Association [14] and explained how to use the app and all study instructions. In this study, we utilized a pre-existing diabetes management app. In addition, we upgraded a function to link the app data to the providers’ main server. All participants in the intervention group were provided with a glucometer (GlucoNavii SD GlucoLink 0.3, SD Biosensor Inc) and 100 strips. Participants who satisfied all three of the following criteria were additionally provided with an electronic manometer (BP-1209, YH Medical Co): with hypertension for more than 1 year, taking hypertension medication for more than 3 months, and systolic or diastolic BP ≥140/90 at the screening.

Participants in the intervention group were required to upload the daily self-monitoring of blood glucose (SMBG) results using the mobile phone app for 3 months (Multimedia Appendix 2). As a coaching hospital center, we provided the general guidelines for SMBG measurements to all physicians in the primary care clinics. Because there is evidence that supports a correlation between higher SMBG frequency and lower HbA_1c_ [15,16], we established SMBG measurement guidelines to encourage more SMBG testing when the patient’s HbA_1c_ level was high. Therefore, the minimum required number of SMBG measurements was determined on the basis of the participant’s baseline HbA_1c_ level. Participants were advised to check the SMBG twice weekly when their HbA_1c_ level was <7% (53 mmol/mol) without oral hypoglycemic agents (OHAs); once daily when their HbA_1c_ level was <7% (53 mmol/mol) with OHA; at least once daily when their HbA_1c_ was between 7% to 8% (53-64 mmol/mol) with OHA or was >8% (64 mmol/mol) regardless of OHA; twice daily when their HbA_1c_ level was <7% (53 mmol/mol) with insulin treatment; and thrice daily when their HbA_1c_ level was ≥7% (53 mmol/mol) with insulin treatment. When participants checked their SMBG with the provided glucometer and brought it into contact with the mobile phone, the SMBG result was automatically inputted into the mobile phone app, using wireless transmission through a near-field communication system. Participants could also upload the data via manual input. The mobile phone then automatically transmitted the data to the main server. Participants were also required to input the mealtime when SMBG was checked and were requested to input their BP at least once a week. They could also input weight data on the mobile phone app.

The physicians of the primary care clinic had to check the accumulated participants’ data and send short feedback messages via a password-protected staff website at least once a week. As a coaching hospital center, we provided a manual on how to recommend messages to each clinic. We also provided some examples of message templates in the administrator’s website, where the physicians could select and send a message. The medical staff could select or modify the example template as desired. The main contents of the message were about praise or encouragement if participants’ SMBG was almost within the target level, a reinformation of their glucose target level or advice for dietary control and exercise if their SMBG was almost above the target level, or an advice for the regular glucose checkup if they did not check the SMBG at the recommended level. Advice about dietary control and exercise was based on general guidelines such as reduce the carbohydrate and fruit intake and encourage postmeal exercise. If necessary, physicians could conduct additional direct phone call consultations, although it was not mandatory.

Every month, all study participants of the intervention and control groups visited the outpatient clinic and received face-to-face consultations for individual management target of risk factors (Multimedia Appendix 2). At this time, the physicians of the intervention clinic additionally provided the summarized result of accumulated information from the staff website to their patients and gave a consultation based on it.

### Measures

All study participants in the intervention or control group received a baseline assessment for demographic information regarding age, sex, alcohol intake, cigarette smoking, past history, and medications. Baseline measurements of height, weight, waist circumference (WC), and BP were also conducted. Height and weight were measured in light clothing without shoes. An experienced nurse measured the WC with the participant standing erect with his or her arms at the side, keeping their feet wide open about 15 cm. BMI was calculated using the participant’s height and weight (kg/m^2^). BP was measured in the sitting position by the oscillometric method using an appropriate cuff after resting at least 5 min. A baseline fasting blood sample was obtained to measure the fasting plasma glucose (FPG), HbA_1c_, creatinine, AST, ALT, total cholesterol, triglyceride, high-density lipoprotein (HDL) cholesterol, and low-density lipoprotein cholesterol. All blood samples were transported to a central laboratory (Green Cross Laboratories) for analysis. HbA_1c_ was measured through high-performance liquid chromatography.

Each monthly visit, physicians checked participants’ vital signs, weight, WC, change in medications, and assessed safety and compliance with the study. At baseline and 3 months, all participants underwent the same measurements of anthropometry and laboratory tests to evaluate the efficacy of the trial.

### Questionnaires

The questionnaires were given to the subjects twice, at baseline and 3 months, to evaluate participants’ treatment satisfaction and adherence to chronic medications. The participants received the surveys in written form and self-conducted it in a separate clinic space. If they had difficulty in filling out the questionnaires, the primary care provider could give them help. Finally, the questionnaires were collected and analyzed by research statistician not involved with this study.

The Diabetes Treatment Satisfaction Questionnaire status version (DTSQs) was used to evaluate the participants’ treatment satisfaction [17]. DTSQs contains 8 items scored on a 7-point scale from 6 (extremely satisfied) to 0 (very dissatisfied) points. Higher total scores indicate favorable treatment satisfaction. The 6-item Morisky Medication Adherence Scale (MMAS-6) was used to evaluate participants’ motivation and knowledge related to long-term medication adherence in chronic diseases [18]. The response categories are yes or no for each item, with 1 point given to the desired state. If the total score for questions 1, 2, and 6 equals 0 or 1, this means that they have low motivation, whereas a total score of 2 or 3 refers to high motivation for medication adherence. If the total score for questions 3, 4, and 5 equals 0 or 1, this means that they have low knowledge of medication adherence, and a total score of 2 or 3 refers to the high level of knowledge.

### Outcomes

The primary efficacy outcome was the difference in mean change in HbA_1c_ from baseline to the 3-month follow-up between the two groups. The secondary efficacy outcomes were the difference of mean change from baseline to 3 months in FPG, weight, WC, BMI, systolic and diastolic BP, lipid profile, and the DTSQs and MMAS-6 questionnaire scores between the two groups.

Any adverse events were coded using the Medical Dictionary for Regulatory Activities, regarding the intervention, and were assessed by the medical staff at follow-up visits or by patient self-reporting.

### Statistical Analysis

Calculation of the sample size was based on an expected 0.6% difference of change in HbA_1c_ (primary outcome) level between the intervention and control groups, with an SD of 0.8, an average cluster size of 25, and an intracluster correlation coefficient of .10, similar to a previous study [19]. As a result, a target sample of 100 participants per each group would achieve 80% power at a critical significance level of 0.05. A higher dropout rate was expected in the intervention group, so we planned to enroll more clinics and participants in the intervention group. Finally, we planned to enroll 150 participants at 9 intervention clinics and 100 participants at 4 control clinics.

The data were analyzed on an intent-to-treat basis of all assigned participants who completed the follow-up assessment at 3 months. For comparison of baseline differences between the two groups, independent *t* tests for continuous variables and chi-square tests for categorical variables were used. Continuous variables were expressed as means and SD or mean (95% CI). Categorical variables were expressed as numbers (%). To compare within-group differences (mean change from baseline) between baseline and 3 months, differences between pre and postintervention were examined using a paired *t* test. Analysis of covariance (ANCOVA) was used to compare mean changes in primary and secondary efficacy outcomes between the control and intervention groups (adjusted mean difference to control). Results were assessed using ANCOVA with a fixed effect for intervention, and age and respective baseline value as covariates to calculate a least-squares estimate of the treatment difference.

Furthermore, sensitivity analyses were assessed using the linear mixed model with fixed effects for age, sex, intervention, time (baseline and 3 months), respective baseline value, baseline value-by time interaction and intervention-by time interaction, and random effects with cluster (centers) and each participant. All analyses were two-tailed, and clinical significance was defined as *P*<.05. Statistical analyses were performed with the statistical package SPSS version 24.0 (SPSS Inc, Chicago, Illinois, the United States) or SAS version 9.3.

## Results

### Participant Disposition and Characteristics

**Figure 1:**
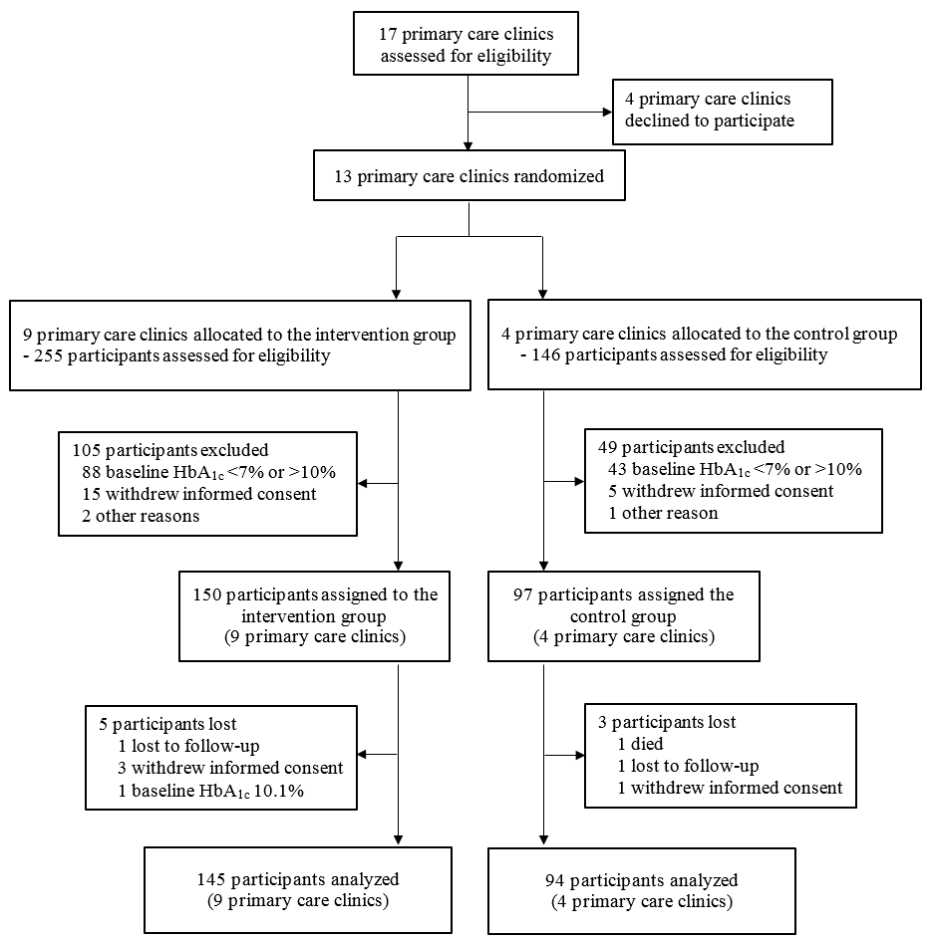
Study enrollment and follow-up.

Between March and June 2015, a total of 401 subjects were assessed for eligibility: 255 subjects at 9 intervention clinics and 146 subjects at 4 control clinics (Figure 1). Among them, 150 subjects at intervention clinics and 97 subjects at control clinics met all the criteria and participated in the study. Only 5 and 3 subjects in intervention and control clinics were lost during follow-up, respectively. No clinic stopped participating. Therefore, 239 final subjects were analyzed in this study: 145 subjects at 9 intervention clinics and 94 subjects at 4 control clinics.

The baseline characteristics of the participants in both groups are shown in Table 1. Both groups showed similar sex distributions. However, the mean age of the intervention group was 54.1 years, which was significantly lower than the 60.6 years of the control group. Although there were no significant differences in BMI and proportion of obesity (BMI ≥25 kg/m^2^) between the 2 groups, the intervention group showed higher weight and WC compared with those in the control group. The subjects in the intervention group also had higher diastolic BP at baseline. The baseline HbA_1c_ was similar between the 2 groups, and mean HbA_1c_ was 7.9% in the intervention and 8.0% in the control group, respectively. In addition, baseline laboratory data showed almost no statistical differences between the 2 groups, except for the lower total cholesterol and HDL cholesterol levels in the intervention group. Baseline characteristics by each clinic in the control and intervention groups are shown in Multimedia Appendices 3 and 4.

**Table 1:** Baseline characteristics of study participants.

Variable		Control group (n=97)	Intervention group (n=150)	*P* value
**Age (years)**				<.001
	Mean (SD)	60.6 (10.2)	54.1 (10.1)	
	<40, n (%)	4 (4)	10 (6.7)	
	≥40 and <60, n (%)	36 (37)	93 (62.0)	
	≥60, n (%)	57 (59)	47 (31.3)	
Male, n (%)		45 (46)	80 (53.3)	.29
Height (cm), mean (SD)		161.3 (9.1)	163.6 (9.5)	.06
Weight (kg), mean (SD)		67.2 (14.2)	70.6 (12.8)	.005
**BMI (kg/m ^**2**^)**				
	Mean (SD)	25.7 (3.9)	26.3 (3.7)	.17
	Obesity (BMI≥25), n (%)	52 (54)	97 (64.7)	.08
Waist circumference (cm), mean (SD)		87 (9.8)	89.5 (8.9)	.003
Systolic BP^a^ (mmHg), mean (SD)		124.5 (11.9)	126.3 (10.9)	.17
Diastolic BP (mmHg), mean (SD)		74.1 (10.3)	77.3 (9)	.009
Diagnosis of hypertension, n (%)		57 (59)	92 (61.3)	.69
Diagnosis of dyslipidemia, n (%)		62 (64)	108 (72.0)	.18
Current smoker, n (%)		18 (19)	31 (20.7)	.69
FPG^b^ (mg/dL), mean (SD)		147.9 (48.7)	150.7 (57.2)	.95
**HbA_1c_^c^**(%)****				
	Mean (SD)	7.9 (0.8)	8 (0.8)	.52
	≥8%, n (%)	38 (39)	66 (44.0)	.45
Total cholesterol (mg/dL), mean (SD)		165 (30.5)	156.6 (29.8)	.02
Triglyceride (mg/dL), mean (SD)		165.3 (81.1)	160.3 (106.1)	.08
HDL^d^ cholesterol (mg/dL), mean (SD)		51.1 (13.5)	46.9 (11)	.01
LDL^e^ cholesterol (mg/dL), mean (SD)		94.5 (26.6)	89.6 (26.1)	.22
AST^f^ (U/L), mean (SD)		25.9 (13)	27.4 (17.5)	.86
ALT^g^ (U/L), mean (SD)		26.2 (15)	30.6 (22.5)	.16
Serum creatinine (mg/dL), mean (SD)		0.9 (0.2)	0.8 (0.2)	.09
DTSQs^h^ score, mean (SD)		27.6 (6.1)	28.2 (6.2)	.35
**MMAS-6^i^** **score, mean (SD)**				
	Total	4.7 (1.1)	4.4 (1.3)	.16
	Motivation	2.3 (0.9)	2 (1)	.01
	Knowledge	2.3 (0.6)	2.4 (0.7)	.09

^a^BP: blood pressure.

^b^FPG: fasting plasma glucose.

^c^HbA_1c_: hemoglobin A_1c_.

^d^HDL: high-density lipoprotein.

^e^LDL: low-density lipoprotein.

^f^AST: aspartate transaminase.

^g^ALT: alanine transaminase.

^h^DTSQs: Diabetes Treatment Satisfaction Questionnaire status version.

^i^MMAS-6: 6-item Morisky Medication Adherence Scale.

### Changes in Glycemic Status

Table 2 shows the change in efficacy outcomes compared with baseline between the two groups at 3 months. The mean changes in efficacy outcomes from baseline in each clinic of the control and intervention groups are shown in Multimedia Appendices 5 and 6. At 3 months, both groups showed significant decreases in HbA_1c_ level compared with the baseline. Nevertheless, the intervention group showed significantly more reduction in HbA_1c_ compared with the control group, and the adjusted mean difference of change in HbA_1c_ between the two groups was −0.30% (95% CI −0.50% to −0.11%). The proportions of subjects who achieved HbA_1c_ <7% (53 mmol/mol) at 3 months were 33.8% (49/145) in the intervention and 24% (23/94) in the control group, respectively. Similar results were observed in the FPG level in both groups. The intervention group showed significantly more reduction in FPG (−7.29 mg/dL, 95% CI −29.33 to −5.26) than the control group. Sensitivity analysis showed constant statistical significance in all outcomes of HbA_1c_ and FPG.

**Table 2:** Changes in efficacy outcomes between control and intervention groups at 3 months.

Outcomes			Mean change from baseline				Adjusted mean difference to control^a^, mean (95% CI)		*P* value^a^		*P* value for interaction (intervention x time)^b^	
			Control group (n=94), mean (95% CI)		Intervention group (n=145), mean (95% CI)							
**Glycemic** **parameters**												
	HbA_1c_^c^ (%)			−0.28 (−0.42 to −0.13)		−0.63 (−0.77 to −0.50)		−0.30 (−0.50 to −0.11)		.003^d^		.001^d^
	HbA_1c_ (mmol/mol)			−3.02 (−4.62 to −1.42)		−6.93 (−8.38 to −5.48)		−3.32 (−5.50 to −1.15)		.003^d^		.001^d^
	FPG^e^ (mg/dL)			−2.41(−13.64 to 8.82)		−19.11 (−29.80 to −8.43)		−17.29 (−29.33 to −5.26)		.005^d^		.02^d^
**Other metabolic parameters**												
	Weight (kg)			−0.88 (−2.65 to 0.90)		−0.63 (−1.02 to −0.24)		0.22 (−1.26 to 1.71)		.77		.35
	WC^f^ (cm)			−0.88 (−1.61 to −0.16)		−0.93 (−1.46 to −0.40)		0.30 (−0.62 to 1.22)		.52		.76
	BMI (kg/m^2^)			−0.41 (−1.21 to 0.40)		−0.26 (−0.40 to −0.11)		0.09 (−0.48 to 0.65)		.77		.34
	Systolic BP^g^ (mmHg)			3.55 (1.30 to 5.81)		−0.20 (−2.30 to 1.90)		−3.66 (−6.57 to −0.76)		.01^d^		.003^d^
	Diastolic BP (mmHg)			0.68 (−0.94 to 2.30)		−2.02 (−3.47 to −0.57)		−2.77 (−4.92 to −0.62)		.01^d^		.08
	Total cholesterol (mg/dL)			−2.77 (−8.01 to 2.48)		−3.06 (−6.73 to 0.60)		−3.81 (−10.04 to 2.42)		.23		.35
	Triglyceride (mg/dL)			−16.88 (−30.14 to −3.62)		−16.72 (−31.36 to −2.08)		−8.27 (−25.89 to 11.27)		.38		.73
	HDL^h^ cholesterol (mg/dL)			0.24 (−1.35 to 1.84)		2.44 (1.16 to 3.73)		1.40 (−0.45 to 3.39)		.15		.20
	LDL^i^ cholesterol (mg/dL)			−0.16 (−4.60 to 4.28)		−2.99 (−6.03 to 0.04)		−4.46 (−9.62 to 0.69)		.09		.10
**Questionnaires^j^**												
	DTSQs^k^			0.45 (−1.03 to 1.92)		2.40 (1.22 to 3.58)		2.21 (0.54 to 3.88)		.01^d^		.04^d^
	**MMAS-6^l ^**			0.06 (−0.15 to 0.28)		0.52 (0.31 to 0.74)		0.31 (0.05 to 0.57)		.02^d^		.02^d^
		Motivation		0.04 (−0.11 to 0.20)		0.39 (0.23 to 0.54)		0.23 (0.03 to 0.42)		.02^d^		.01^d^
		Knowledge		0.02 (−0.13 to 0.17)		0.14 (0.00 to 0.28)		0.12 (−0.03 to 0.28)		.12		.08

^a^Assessed using the analysis of covariance model with a fixed effect for intervention, and age and respective baseline value as covariates to calculate a least-squares estimate of the treatment difference.

^b^Assessed using the linear mixed model with fixed effects for age, sex, intervention, time (baseline and 3 months), respective baseline value, baseline value-by time interaction, intervention-by time interaction, and random effects with cluster (centers) and each participant.

^c^HbA_1c_: hemoglobin A_1c_.

^d^*P*<.05.

^e^FPG: fasting plasma glucose.

^f^WC: waist circumference.

^g^BP: blood pressure.

^h^HDL: high-density lipoprotein.

^i^LDL: low-density lipoprotein

^j^Higher DTSQs and MMAS-6 scores indicate a favorable state.

^k^DTSQs: Diabetes Treatment Satisfaction Questionnaire status version.

^l^MMAS-6: 6-item Morisky Medication Adherence Scale.

### Effect on Other Metabolic Parameters Including Weight-Related Outcomes, Blood Pressure, and Dyslipidemia

During the 3-month follow-up, both groups showed different changes in various anthropometries and lipid parameters (Table 2). Although only the intervention group showed significant favorable changes in weight and HDL cholesterol compared with baseline, there were no statistically significant differences in the change of overall weight-related and lipid profile outcomes between the 2 groups. In contrast, the intervention group showed significantly more reduction in systolic and diastolic BP than the control group. However, with the sensitivity analysis, the statistical significance was lost in the change of diastolic BP.

### Diabetes Treatment Satisfaction Questionnaire Status Version and 6-Item Morisky Medication Adherence Scale

DTSQs showed a significant rise only in the intervention group, resulting in a 2.21-point increase in the intervention group compared with the control group at 3 months (Table 2). Total MMAS-6 score was also significantly more increased in the intervention group, especially in the score related to motivation for long-term medication adherence. However, the knowledge aspect showed little difference between the two groups.

### Subgroup Analyses of Changes in the Glycemic Status

**Figure 2:**
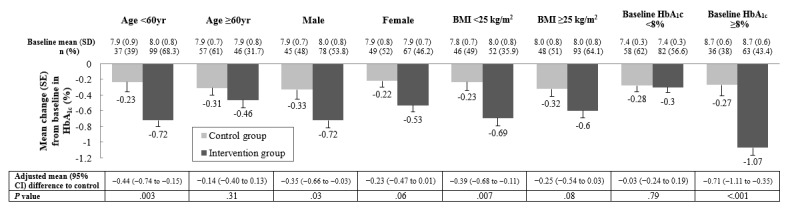
Subgroup analyses of changes in glycemic status by age, sex, BMI, and baseline hemoglobin A_1c_ (HbA_1c_). Baseline data are expressed as mean (SD), and mean change of outcomes are expressed as mean (SE). The gray and black bars represent the control and intervention groups, respectively.

We conducted subgroup analyses of the changes in glycemic status according to participant age, sex, BMI, and baseline HbA_1c_ level (Figure 2). Because of the difference in the baseline average age between the 2 groups, we divided each group into 2 subgroups: age <60 and ≥60 years. In the analysis of participants under 60 years of age, there were significantly reduced HbA_1c_ and FPG levels (Multimedia Appendix 7) in the intervention group, compared with those in the control group. However, there was little difference in these changes between the two groups in the analysis of participants 60 years and older.

In the subgroup analyses, according to sex, men showed significantly more reduction in HbA_1c_ level, whereas women showed significantly more reduction in FPG level compared with the control group. In the subgroup analyses according to BMI, the nonobese group (BMI <25 kg/m^2^) showed significantly more reduction in HbA_1c_ level, whereas obese group (BMI ≥25 kg/m^2^) showed significantly more reduction in FPG level compared with the control group.

Among participants with baseline HbA_1c_ levels lower than 8%, the intervention group did not show a difference in HbA_1c_ reduction compared with the control group, despite more reduction in FPG. However, among participants with baseline HbA_1c_ levels of 8% and higher, the intervention group showed the most significant reduction in HbA_1c_ and FPG compared with the modest change observed in the control group.

### Changes in Glycemic Status According to Self-Monitoring of Blood Glucose Compliance

**Figure 3:**
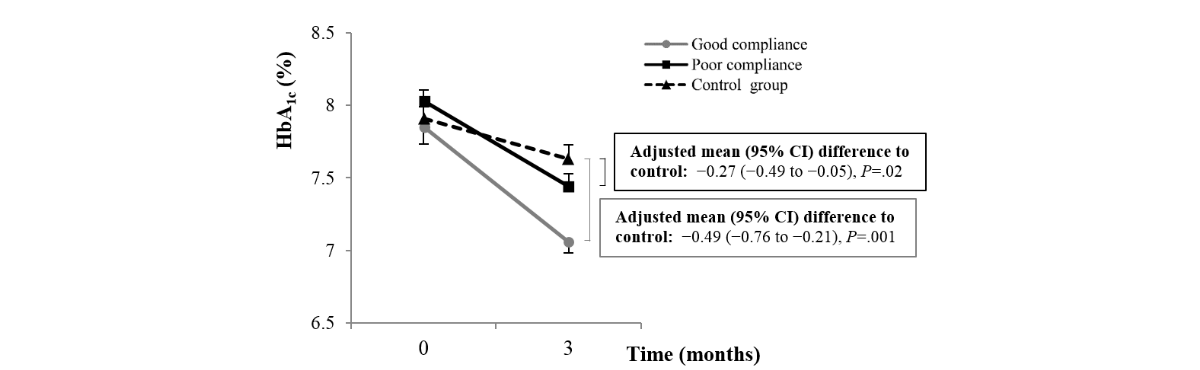
Changes in hemoglobin A_1c_ (HbA_1c_) according to the self-monitoring of blood glucose compliance of the intervention group compared with the control group. Data are expressed as mean (SE).

Figure 3 shows the changes in HbA_1c_ levels according to good and poor compliance with SMBG in the intervention group, compared with the control group. Compliance was calculated as the mean SMBG count/day (total numbers of SMBG count during the total study period divided by the study days for each participant). Participants with good compliance were defined as those with mean SMBG count/day ≥1, whereas those with SMBG count/day <1 were defined as having poor compliance. As a result, the participants with good compliance showed a higher reduction in HbA_1c_ than participants with poor compliance, although both good and poor compliance participants showed significantly more reduction in HbA_1c_ compared with the control group. In contrast, participants with good compliance only showed significantly more reduction in FPG than the control group, compared with the little change between the poor compliance group and the control group (Multimedia Appendix 8).

### Adverse Events

During the study, 12.4% (18/145) participants in the intervention group and 8% (8/94) participants in the control group reported a variety of symptoms, which were classified as adverse events (Table 3). The numbers of any adverse events did not differ significantly between the two groups. The most frequent events were elevated liver enzymes and hypoglycemia, which occurred only in the intervention group. However, all the reported symptoms were mild and temporary.

One participant in the control group died from cerebral infarction, which was classified as serious adverse events during the study period. However, there was no identified causality associated with the intervention system.

**Table 3:** Adverse events through 3 months.

Adverse events			Control group (n=94), n (%)	Intervention group (n=145), n (%)	*P* value
**Any adverse events**			8 (9)	18 (12.4)	.35
	Elevated liver enzymes		5 (5)	12 (8.3)	
	Renal impairment		2 (2)	1 (0.7)	
	Mild hypoglycemia		0 (0)	5 (3.5)	
	**Serious adverse events**				
		Death	1 (1)^a^	0 (0)	

^a^Death from cerebral infarction.

## Discussion

### Principal Findings

In this study, we demonstrated the clinical effect of a mobile phone–based diabetes management system in multiple primary care clinics. Participants in the intervention group showed significantly more reduction in HbA_1c_ and FPG levels at 3 months compared with that of the control group. In addition to the improvement of glycemic status, there was also a significantly more reduction in BP and more improvement of participants’ satisfaction and motivation for the management of the chronic disease. With the subgroup analyses, the effect on glycemic control was more significant in those with good compliance with SMBG, younger age (<60 years), and with poor glycemic status at baseline (HbA_1c_ ≥8%).

With the developments in IT, the method of monitoring glucose level has become simpler and more convenient for both patients with diabetes and their physicians [20]. There has been much evidence of the effects of Web- and mobile phone–based glucose-monitoring systems on diabetes management [21,22]. Nevertheless, most of the previous studies targeted patients under the care of tertiary hospitals and provided limited evidence in primary care clinic settings. Tertiary hospitals generally have a sufficient workforce, including professional diabetes specialists and skilled assistant medical teams. Also, with abundant educational experiences and professional equipment, the desirable and intended management could be easily achieved. However, the use of mobile phone–based diabetes management systems should spread to primary care clinics where these resources are insufficient. A previous study of a Web-based glucose-monitoring and feedback system showed that short motivational feedback messages, such as encouragement or simple recommendations, rather than complicated ones, were effective in glycemic control [23]. Thus, despite the lack of experience and resources, the use of mobile phone–based management systems at primary care clinics was likely to have a similar effect as tertiary hospitals.

In this study, the role of an experienced tertiary hospital was limited to the coaching center, and most clinics could use the system independently without much difficulty. As a result, there was no drop-out at participating clinics during the study period. At 3 months, we observed more statistically significant improvements in HbA_1c_ and FPG levels in the intervention group than in the control group, which demonstrates the clinical effectiveness and applicability of this mobile health system in the primary care environment. The mean difference between intervention and control groups was −0.30%, which is slightly lower than that reported by a meta-analysis of tertiary hospitals (−0.4 to −0.67%) [22,24], and slightly higher than that reported in a recent Web-based study targeting primary care clinics in England (−0.24%) [25]. Interestingly, most of the intervention clinics showed similar results of glycemic improvement in the outcome analysis by each clinic, except for one intervention clinic (Multimedia Appendix 6). Although we could not precisely evaluate the physician’s interaction with the participant in this study, it could be a determinant factor for the effective usage of this system [26]. Besides, participants with good compliance with SMBG showed more improvement in HbA_1c_ as in previous studies [27,28], and the mean difference in HbA_1c_ change was −0.49%. In this study, among the 145 participants in the intervention group, only 34 (23.4%) were classified as good compliance with SMBG. For a more effective application to primary care clinics, additional strategies for the improvement of compliance, such as a more accessible system or noninvasive methods for SMBG, are needed to provide positive results [29]. The questionnaire survey showed greater favorable changes in the satisfaction and motivation aspects for long-term medication adherence in the intervention group than in the control group. Previous studies reported the increased effectiveness of mobile phone–based management systems according to higher satisfaction and medication adherence [30,31]. These positive results are thought to have affected the positive effect of this study.

Interestingly, after the 3-month intervention, the intervention group also showed significantly more decrease in BP, along with improvement of glycemic status. Our intervention strategies for BP were limited to providing an electronic manometer to the participants with proven hypertension, and we recommended inputting their BP data at least once a week. In comparison, other metabolic parameters, including weight-related outcomes and dyslipidemia, did not show any effect. At baseline, the lipid profiles of both groups were controlled relatively well, and most participants were already taking statins. In addition, the input of weight data was not mandatory in the study. As a result, the impact on weight and lipid levels is thought to have been minimal. In the management of diabetes, it is essential to control various combined metabolic risk factors together to maximize the risk reduction of diabetic complications [32,33]. Therefore, more strategies for weight-related outcomes will need to be supplemented in the following studies.

Subgroup analyses showed little gender and BMI difference in glucose reduction. However, the effect on glycemic control was limited only to those under the age of 60 years. Since the study showed a baseline difference in age between the 2 groups, this finding suggests that our intervention effect in the reduction of HbA_1c_ and FPG was mostly attributable to participants under the age of 60 years. There is much evidence showing the effectiveness of Web- and mobile phone–based systems in elderly patients [10,34,35]. The reason why the mobile phone–based system had less impact on elderly patients in this study may be due to the lower socioeconomic and educational status of primary care patients compared with tertiary hospitals, which might be the particular situation in South Korea where the barriers to tertiary hospitals are lower than those of other countries [36]. In addition, subgroup analyses of baseline HbA_1c_ levels revealed a more significant reduction among participants with baseline HbA_1c_ levels of 8% and over. However, the difference in HbA_1c_ reduction between the control and the intervention groups was not observed in participants with baseline HbA_1c_ levels lower than 8%. In the previous study on the long-term effects of a Web-based glucose-monitoring system in patients with well-controlled diabetes (baseline HbA_1c_ <7%), the intervention group remained stable throughout the study with a low fluctuation of HbA_1c_ level, compared with the high fluctuation in the control group [23]. Therefore, expanded study periods are needed to evaluate long-term effects, especially in patients with well-controlled glycemic status.

### Limitations

Our study has several limitations. First, as discussed previously, there was a significant age difference at baseline between the intervention and control groups, despite the randomized setting. The younger age of the intervention group may have a significant influence on compliance, providing more positive findings. On the basis of baseline characteristics by each clinic (Multimedia Appendices 3 and 4), we found that younger participants were mostly enrolled in intervention clinics compared with the control clinics. It might be because many of those who voluntarily participated in intervention clinics had more interest in the IT-based intervention and were younger than those in control clinics. Second, as a cluster-randomized open-label trial, variations in each medical team’s interventions could cause bias. Finally, the follow-up duration was short, and only primary care clinics in large cities were targeted. Further studies targeting the primary care clinics in rural areas, where resources are more insufficient, and longer study periods are needed to provide extended evidence of mobile phone–based diabetes management systems. However, our research has the strength of being a well-designed and well-proceeded multicenter cluster-randomized controlled study with a relatively large number of clinics and participants. With a cluster-randomized setting, we could minimize the treatment contamination—a critical issue of educational intervention—between intervention and control participants [37].

### Conclusions

In this study, we confirmed the clinical efficacy and applicability of a mobile phone–based diabetes-monitoring and feedback system in primary care clinics, which relatively lack the professional workforce and educational environment for chronic disease management. Younger patients with poor glycemic status (HbA_1c_ ≥8%) and good compliance with SMBG are those who may benefit the most from this intervention.

## Appendix

Multimedia Appendix 1 The 13 private clinics that participated in this study.

Multimedia Appendix 2 Study design.

Multimedia Appendix 3 Baseline characteristics by each clinic in the control group.

Multimedia Appendix 4 Baseline characteristics by each clinic in the intervention group.

Multimedia Appendix 5 Mean changes in efficacy outcomes from baseline in each clinic of the control group.

Multimedia Appendix 6 Mean changes in efficacy outcomes from baseline in each clinic of the intervention group.

Multimedia Appendix 7 Subgroup analyses of changes in fasting plasma glucose by sex, age, BMI, and baseline hemoglobin A1c.

Multimedia Appendix 8 Changes in fasting plasma glucose according to the self-monitoring of blood glucose compliance of the intervention group compared with the control group.

Multimedia Appendix 9 CONSORT EHEALTH checklist (V 1.6.1).

## Abbreviations

ALT: alanine aminotransferase
ANCOVA: analysis of covariance
AST: aspartate aminotransferase
BP: blood pressure
DTSQs: Diabetes Treatment Satisfaction Questionnaire status version
FPG: fasting plasma glucose
HbA_1c_: hemoglobin A_1c_
HDL: high-density lipoprotein
ICT: Information and Communications Technology
IT: information technology
LDL: low-density lipoprotein
MMAS-6: 6-item Morisky Medication Adherence Scale
NRF: National Research Foundation
OHA: oral hypoglycemic agents
SMBG: self-monitoring of blood glucose
T2DM: type 2 diabetes mellitus
WC: waist circumference
