# Supplementary material for: Effect of a Mobile Phone–Based Glucose-Monitoring and Feedback System for Type 2 Diabetes Management in Multiple Primary Care Clinic Settings: Cluster Randomized Controlled Trial
Source: JMIR Mhealth Uhealth. 2020 Feb 26;8(2):e16266. doi: 10.2196/16266 (PMC7066511; doi:10.2196/16266)
Supplement: Multimedia Appendix 1 [file mhealth_v8i2e16266_app1.docx]

**Multimedia Appendix 1**

The 13 private clinics that participated in this study.

| Group | Site number | Primary care clinics | Participating investigators | City |
| --- | --- | --- | --- | --- |
| Intervention clinic group | 01 | Dream Saint Mary's Internal Medicine Clinic | Sinae Park | Suwon |
|  | 02 | Seoul Saint Mary's Internal Medicine Clinic | Seokju Lee | Bucheon |
|  | 04 | Yeil Saint Mary's Internal Medicine Clinic | Hyeonsu Kim | Seoul |
|  | 05 | Mercy IM Clinic | Giyeong Kim | Incheon |
|  | 06 | Heo Joon's Medical Clinic | Joon Heo | Cheonan |
|  | 09 | Bupyung Medical Center | **Gyeonggeun Koh** | Incheon |
|  | 10 | Cheon An ENDO Medical Clinic | **Seokgi Yoon** | Cheonan |
|  | 13 | Seoul MB Internal Medicine Clinic | **Yeonggeun Hyun** | Seoul |
|  | 16 | Kim Medicine | **Jaeyeong Kim**, **Junil** **Park, Jiwon Ahn** | Seoul |
| Control clinic group | 03 | HAN's Medical Clinic | **Chungmin Han** | Namyangju |
|  | 07 | Moon Chan Soo Internal Medicine Clinic | Chan Soo Moon | Seoul |
|  | 12 | Opened Saint Mary's Internal Medicine Clinic | **Hyeonjae** Kim | Incheon |
|  | 17 | Huh Diabetes Clinic | **Gapbeom** **Huh,** **Yeongju Choi** | Seoul |
