# Supplementary material for: Effect of a Mobile Phone–Based Glucose-Monitoring and Feedback System for Type 2 Diabetes Management in Multiple Primary Care Clinic Settings: Cluster Randomized Controlled Trial
Source: JMIR Mhealth Uhealth. 2020 Feb 26;8(2):e16266. doi: 10.2196/16266 (PMC7066511; doi:10.2196/16266)
Supplement: Multimedia Appendix 3 [file mhealth_v8i2e16266_app3.docx]

**Multimedia Appendix 3**

Baseline characteristics by each clinic in the control group.

| Variable | | Site number of control clinic group | | | |
| --- | --- | --- | --- | --- | --- |
|  |  | 03  (n=29) | 07  (n=18) | 12  (n=20) | 17  (n=30) |
| Age (years) | |  |  |  |  |
|  | Mean (SD) | 62.6 (8.6) | 58.1 (12.6) | 59.3 (10.5) | 61.0 (9.8) |
|  | <40, n (%) | 0 (0) | 2 (11) | 1 (5) | 1 (3) |
|  | ≥40 and <60, n (%) | 10 (34) | 7 (39) | 7 (35) | 12 (40) |
|  | ≥60, n (%) | 19 (66) | 9 (50) | 12 (60) | 17 (57) |
| Male, n (%) | | 13 (45) | 11 (61) | 12 (60) | 11 (37) |
| Height (cm), mean (SD) | | 158.8 (9.5) | 165.8 (8.5) | 162.9 (8.6) | 159.9 (8.7) |
| Weight (kg), mean (SD) | | 70.7 (18.9) | 70.1 (17.0) | 70.1 (21.2) | 62.6 (7.6) |
| BMI (kg/m^2^) | |  |  |  |  |
|  | Mean (SD) | 28.1 (8.2) | 25.3 (4.0) | 26.1 (5.8) | 24.5 (2.9) |
|  | Obesity (BMI ≥25), n (%) | 20 (69) | 10 (56) | 10 (50) | 12 (40) |
| Waist circumference (cm), mean (SD) | | 86.9 (8.2) | 84.9 (11.2) | 91.6 (12.6) | 84.7 (6.3) |
| Systolic BP^a^ (mmHg), mean (SD) | | 126.4 (10.1) | 124.6 (6.6) | 127.5 (9.7) | 121.7 (11.2) |
| Diastolic BP (mmHg), mean (SD) | | 78.4 (9.2) | 73.9 (6.9) | 78.7 (6.4) | 67.0 (9.9) |
| Diagnosis of hypertension, n (%) | | 19 (66) | 8 (44) | 14 (70) | 16 (53) |
| Diagnosis of dyslipidemia, n (%) | | 16 (55) | 14 (78) | 12 (60) | 20 (67) |
| Current smoker, n (%) | | 2 (7) | 6 (33) | 6 (30) | 4 (13) |
| FPG^b^ (mg/dL), mean (SD) | | 141.7 (45.2) | 183.1 (71.0) | 136.5 (43.2) | 141.6 (30.1) |
| HbA_1c_^c^ (%) | |  |  |  |  |
|  | Mean (SD) | 7.9 (0.7) | 8.0 (0.7) | 8.1 (0.9) | 7.9 (0.7) |
|  | ≥8%, n (%) | 9 (31) | 10 (56) | 9 (45) | 10 (33) |
| Total cholesterol (mg/dL), mean (SD) | | 162.6 (26.1) | 160.6 (33.1) | 170.7 (31.4) | 166.3 (33.0) |
| Triglyceride (mg/dL), mean (SD) | | 187.5 (73.4) | 190.4 (68.0) | 138.8 (69.6) | 146.4 (94.7) |
| HDL^d^ cholesterol (mg/dL), mean (SD) | | 46.7 (10.7) | 51.2 (17.9) | 55.3 (16.8) | 52.6 (9.2) |
| LDL^e^ cholesterol (mg/dL), mean (SD) | | 91.8 (24.0) | 85.6 (30.3) | 98.8 (25.7) | 96.2 (27.2) |
| AST^f^ (U/L), mean (SD) | | 26.2 (13.6) | 28.2 (14.0) | 23.1 (7.6) | 26.0 (14.8) |
| ALT^g^ (U/L), mean (SD) | | 25.9 (14.0) | 29.9 (15.7) | 23.0 (10.7) | 26.5 (17.8) |
| Serum creatinine (mg/dL), mean (SD) | | 0.9 (0.2) | 0.9 (0.2) | 0.9 (0.2) | 0.9 (0.2) |
| DTSQs^h^ score, mean (SD) | | 27.3 (6.8) | 35.7 (5.0) | 33.7 (7.8) | 29.9 (6.0) |
| MMAS-6^i^ score, mean (SD) | |  |  |  |  |
|  | Total | 4.9 (1.0) | 4.6 (1.0) | 4.5 (1.4) | 4.7 (1.1) |
|  | Motivation | 2.5 (0.8) | 2.6 (0.9) | 2.2 (1.1) | 2.2 (0.8) |
|  | Knowledge | 2.4 (0.6) | 2.0 (0.5) | 2.3 (0.7) | 2.5 (0.6) |

^a^BP: blood pressure.

^b^FPG: fasting plasma glucose.

^c^HbA_1c_: hemoglobin A_1c_.

^d^HDL: high-density lipoprotein.

^e^LDL: low-density lipoprotein.

^f^AST: aspartate transaminase.

^g^ALT: alanine transaminase.

^h^DTSQs: Diabetes Treatment Satisfaction Questionnaire status version.

^i^MMAS-6: 6-item Morisky Medication Adherence Scale.
