# Supplementary material for: Effect of a Mobile Phone–Based Glucose-Monitoring and Feedback System for Type 2 Diabetes Management in Multiple Primary Care Clinic Settings: Cluster Randomized Controlled Trial
Source: JMIR Mhealth Uhealth. 2020 Feb 26;8(2):e16266. doi: 10.2196/16266 (PMC7066511; doi:10.2196/16266)
Supplement: Multimedia Appendix 4 [file mhealth_v8i2e16266_app4.docx]

**Multimedia Appendix 4**

Baseline characteristics by each clinic in the intervention group.

| Variable | | Site number of intervention clinic group | | | | | | | | |
| --- | --- | --- | --- | --- | --- | --- | --- | --- | --- | --- |
|  |  | 01  (n=11) | 02  (n=12) | 04  (n=17) | 05  (n=12) | 06  (n=13) | 09  (n=17) | 10  (n=27) | 13  (n=21) | 16  (n=20) |
| Age (years) | |  |  |  |  |  |  |  |  |  |
|  | Mean (SD) | 51.5 (8.7) | 51.2 (6.0) | 56.5 (9.3) | 52.8 (7.0) | 57.9 (7.3) | 42.0 (8.0) | 54.4 (8.3) | 55.5 (9.5) | 61.5 (11.9) |
|  | <40, n (%) | 1 (9) | 0 (0) | 1 (6) | 0 (0) | 0 (0) | 6 (35) | 0 (0) | 1 (5) | 1 (5) |
|  | ≥40 and <60, n (%) | 8 (73) | 11 (92) | 9 (53) | 10 (83) | 6 (46) | 11 (65) | 19 (70) | 12 (57) | 7 (35) |
|  | ≥60, n (%) | 2 (18) | 1 (8) | 7 (41) | 2 (17) | 7 (54) | 0 (0) | 8 (30) | 8 (38) | 12 (60) |
| Male, n (%) | | 6 (55) | 5 (42) | 9 (53) | 8 (67) | 10 (77) | 10 (59) | 12 (44) | 13 (62) | 7 (35) |
| Height (cm), mean (SD) | | 164.7 (8.4) | 165.4 (8.8) | 164.4 (11.7) | 164.3 (8.2) | 167.1 (9.5) | 167.3 (12.0) | 160.5 (8.2) | 164.0 (8.0) | 159.1 (8.8) |
| Weight (kg), mean (SD) | | 75.9 (11.3) | 75.2 (12.1) | 68.7 (12.8) | 75.1 (12.0) | 75.5 (18.6) | 71.9 (13.8) | 65.8 (11.0) | 65.9 (11.7) | 70.1 (10.8) |
| BMI (kg/m^2^) | |  |  |  |  |  |  |  |  |  |
|  | Mean (SD) | 27.9 (3.8) | 27.5 (4.0) | 25.2 (2.4) | 27.8 (4.3) | 26.8 (4.9) | 25.6 (3.2) | 25.5 (3.3) | 24.3 (2.8) | 27.7 (4.0) |
|  | Obesity (BMI ≥25), n (%) | 8 (73) | 8 (67) | 11 (65) | 10 (83) | 9(69) | 9 (53) | 16 (59) | 11 (52) | 15 (75) |
| Waist circumference (cm), mean (SD) | | 92.9 (11.1) | 89.9 (8.9) | 92.0 (8.5) | 90.3 (8.6) | 91.8 (10.6) | 85.8 (10.5) | 87.5 (8.6) | 86.4 (7.4) | 92.1 (7.5) |
| Systolic BP^a^ (mmHg), mean (SD) | | 126.5 (12.0) | 123.1 (8.4) | 126.0 (12.5) | 126.7 (11.4) | 117.8 (17.9) | 121.5 (11.2) | 122.6 (10.1) | 121.5 (12.1) | 130.1 (6.5) |
| Diastolic BP (mmHg), mean (SD) | | 79.3 (6.7) | 77.1 (5.4) | 82.8 (8.5) | 73.8 (6.9) | 72.8 (11.0) | 77.7 (9.3) | 73.7 (8.1) | 74.0 (8.9) | 79.2 (5.2) |
| Diagnosis of hypertension, n (%) | | 9 (82) | 10 (83) | 8 (47) | 7 (58) | 10 (77) | 7 (41) | 12 (44) | 14 (67) | 15 (75) |
| Diagnosis of dyslipidemia, n (%) | | 5 (45) | 11 (92) | 14 (82) | 5 (42) | 10 (77) | 12 (71) | 19 (70) | 19 (90) | 13 (65) |
| Current smoker, n (%) | | 4 (36) | 3 (25) | 3 (18) | 3 (25) | 2 (15) | 7 (41) | 3 (11) | 5 (24) | 1 (5) |
| FPG^b^ (mg/dL), mean (SD) | | 148.3 (44.6) | 131.2 (42.3) | 141.3 (29.9) | 143.8 (33.0) | 123.3 (21.4) | 144.1 (23.7) | 191.6 (73.0) | 163.3 (95.7) | 131.4 (27.3) |
| HbA_1c_^c^ (%) | |  |  |  |  |  |  |  |  |  |
|  | Mean (SD) | 8.1 (0.8) | 8.1 (0.7) | 7.8 (0.6) | 7.9 (0.5) | 7.6 (0.7) | 7.9 (0.7) | 8.3 (0.9) | 8.1 (1.0) | 8.0 (0.8) |
|  | ≥8%, n (%) | 5 (45) | 7 (58) | 7 (41) | 4 (34) | 2 (15) | 7 (41) | 16 (59) | 8 (38) | 10 (50) |
| Total cholesterol (mg/dL), mean (SD) | | 190.6 (38.6) | 165.6 (38.0) | 155.1 (25.3) | 157.2 (29.5) | 158.0 (28.5) | 145.6 (14.2) | 147.0 (26.8) | 150.5 (28.3) | 160.9 (27.6) |
| Triglyceride (mg/dL), mean (SD) | | 215.8 (120.8) | 145.2 (85.8) | 139.2 (95.0) | 192.6 (104.9) | 116.5 (59.3) | 161.1 (107.4) | 178.7 (133.1) | 151.5 (117.6) | 150.3 (84.2) |
| HDL^d^ cholesterol (mg/dL), mean (SD) | | 42.8 (11.6) | 51.8 (8.1) | 48.0 (11.1) | 41.3 (9.2) | 52.8 (13.8) | 50.3 (12.4) | 44.8 (8.1) | 46.9 (13.2) | 44.5 (8.8) |
| LDL^e^ cholesterol (mg/dL), mean (SD) | | 118.5 (28.8) | 95.2 (31.0) | 89.4 (22.3) | 89.1 (30.4) | 92.8 (26.7) | 76.7 (18.7) | 80.3 (22.2) | 83.2 (18.4) | 98.5 (26.0) |
| AST^f^ (U/L), mean (SD) | | 30.5 (27.7) | 45.6 (35.5) | 24.2 (9.6) | 36.0 (23.6) | 26.6 (10.0) | 25.6 (10.0) | 23.4 (10.8) | 20.1 (9.3) | 27.0 (9.5) |
| ALT^g^ (U/L), mean (SD) | | 28.9 (22.2) | 50.5 (45.8) | 22.6 (9.4) | 48.2 (30.7) | 31.3 (19.3) | 30.9 (18.4) | 27.9 (16.5) | 23.0 (13.8) | 26.6 (13.7) |
| Serum creatinine (mg/dL), mean (SD) | | 0.9 (0.2) | 0.8 (0.2) | 0.8 (0.2) | 0.9 (0.2) | 0.9 (0.2) | 0.8 (0.2) | 0.8 (0.2) | 0.9 (0.2) | 0.8 (0.2) |
| DTSQs^h^ score, mean (SD) | | 31.3 (6.9) | 32.7 (5.0) | 31.4 (5.3) | 29.0 (6.2) | 34.8 (6.1) | 31.4 (5.6) | 34.4 (8.3) | 28.2 (5.6) | 34.8 (8.1) |
| MMAS-6^i^ score, mean (SD) | |  |  |  |  |  |  |  |  |  |
|  | Total | 4.3 (1.1) | 4.3 (1.6) | 4.4 (1.1) | 4.5 (1.8) | 4.8 (1.1) | 4.0 (1.4) | 4.5 (1.4) | 5.0 (1.0) | 4.1 (1.7) |
|  | Motivation | 1.6 (1.2) | 1.9 (1.1) | 2.0 (0.9) | 2.2 (1.1) | 2.5 (0.7) | 1.7 (1.1) | 2.1 (1.0) | 2.3 (0.9) | 1.8 (1.3) |
|  | Knowledge | 2.6 (0.5) | 2.4 (0.9) | 2.4 (0.5) | 2.3 (1.0) | 2.4 (0.9) | 2.3 (0.7) | 2.4 (0.7) | 2.6 (0.5) | 2.4 (0.7) |

^a^BP: blood pressure.

^b^FPG: fasting plasma glucose.

^c^HbA_1c_: hemoglobin A_1c_.

^d^HDL: high-density lipoprotein.

^e^LDL: low-density lipoprotein.

^f^AST: aspartate transaminase.

^g^ALT: alanine transaminase.

^h^DTSQs: Diabetes Treatment Satisfaction Questionnaire status version.

^i^MMAS-6: 6-item Morisky Medication Adherence Scale.
