# Supplementary material for: Effect of a Mobile Phone–Based Glucose-Monitoring and Feedback System for Type 2 Diabetes Management in Multiple Primary Care Clinic Settings: Cluster Randomized Controlled Trial
Source: JMIR Mhealth Uhealth. 2020 Feb 26;8(2):e16266. doi: 10.2196/16266 (PMC7066511; doi:10.2196/16266)
Supplement: Multimedia Appendix 5 [file mhealth_v8i2e16266_app5.docx]

**Multimedia Appendix 5**

Mean changes in efficacy outcomes from baseline in each clinic of the control group.

| Outcomes | | | Mean change from baseline^a^, mean (95% CI) | | | |
| --- | --- | --- | --- | --- | --- | --- |
|  | | | 03  (n=28) | 07  (n=17) | 12  (n=19) | 17  (n=30) |
| Glycemic parameters | | |  |  |  |  |
|  | HbA_1c_^b^ (%) | | −0.47 (−0.73 to −0.21)^c^ | 0.12 (−0.04 to 0.29) | −0.33 (−0.80 to 0.14) | −0.29 (−0.53 to −0.04)^c^ |
|  | HbA_1c_ (mmol/mol) | | −5.11 (−7.96 to −2.27)^c^ | 1.33 (−0.47 to 3.13) | −3.63 (−8.79 to 1.53) | −3.14 (−5.82 to −0.46)^c^ |
|  | FPG^d^ (mg/dL) | | −2.39 (−24.42 to 19.63) | −0.44 (−42.89 to 42.01) | −10.47 (−38.07 to 17.12) | 1.63 (−10.48 to 13.75) |
| Other metabolic parameters | | |  |  |  |  |
|  | Weight (kg) | | −3.26 (−9.30 to 2.79) | 0.66 (−0.72 to 2.05) | 0.29 (−0.45 to 1.03) | −0.27 (−0.69 to 0.16) |
|  | WC^e^ (cm) | | 0.36 (−0.59 to 1.30) | −3.53 (−6.02 to −1.04)^c^ | −0.58 (−2.75 to 1.59) | −0.73 (−1.44 to −0.03)^c^ |
|  | BMI (kg/m^2^) | | −1.45 (−4.19 to 1.29) | 0.19 (−0.24 to 0.62) | 0.13 (−0.15 to 0.40) | −0.10 (−0.28 to 0.07) |
|  | Systolic BP^f^ (mmHg) | | 5.68 (1.00 to 10.36)^c^ | 2.47 (−1.15 to 6.09) | −0.32 (−4.96 to 4.33) | 4.63 (0.04 to 9.22)^c^ |
|  | Diastolic BP (mmHg) | | 2.18 (−0.42 to 4.78) | −0.82 (−4.13 to 2.48) | −1.63 (−5.39 to 2.12) | 1.60 (−1.94 to 5.14) |
|  | Total cholesterol (mg/dL) | | −1.25 (−8.19 to 5.69) | −0.35 (−11.29 to 10.58) | −10.74 (−31.77 to 10.30) | −0.50 (−7.16 to 6.16) |
|  | Triglyceride (mg/dL) | | −28.11 (−52.37 to −3.85)^c^ | 7.53 (−35.07 to 50.13) | −20.53 (−39.48 to −1.57)^c^ | −17.93 (−43.16 to 7.30) |
|  | HDL^g^ cholesterol (mg/dL) | | −0.89 (−2.23 to 4.02) | 1.53 (−1.23, to 4.29) | −0.95 (−6.29 to 4.40) | −0.33 (−2.66 to 1.99) |
|  | LDL^h^ cholesterol (mg/dL) | | 2.43 (−4.17 to 9.03) | −1.59 (−9.54 to 6.37) | −7.68 (−26.00 to 10.63) | 3.00 (−1.62 to 7.62) |
| Questionnaires^i^ | | |  |  |  |  |
|  | DTSQs^j^ | | 3.82 (0.99 to 6.66)^c^ | −2.06 (−5.60 to 1.49) | −2.16 (−5.47 to 1.15) | 0.37 (−2.05 to 2.79) |
|  | MMAS-6^k^ | | −0.14 (−0.57 to 0.29) | 0.00 (−0.75 to 0.75) | 0.26 (−0.19 to 0.71) | 0.17 (−0.14 to 0.48) |
|  |  | Motivation | −0.07 (−0.33 to 0.19) | −0.12 (−0.69 to 0.45) | 0.05 (−0.25 to 0.35) | 0.23 (0.00 to 0.47)^l^ |
|  |  | Knowledge | −0.07 (−0.35 to 0.21) | 0.12 (−0.19 to, 0.43) | 0.21 (−0.13 to 0.55) | −0.07 (−0.36 to 0.23) |

^a^Assessed using the paired *t* test

^b^HbA_1c_: hemoglobin A_1c_.

^c^*P* <.05.

^d^FPG: Fasting plasma glucose.

^e^WC: waist circumference.

^f^BP: blood pressure.

^g^HDL: high-density lipoprotein.

^h^LDL: low-density lipoprotein

^i^Higher DTSQs and MMAS-6 scores indicate a favorable state.

^j^DTSQs: Diabetes Treatment Satisfaction Questionnaire status version.

^k^MMAS-6: 6-item Morisky Medication Adherence Scale.

^l^*P* =.05.
