# Supplementary material for: Effect of a Mobile Phone–Based Glucose-Monitoring and Feedback System for Type 2 Diabetes Management in Multiple Primary Care Clinic Settings: Cluster Randomized Controlled Trial
Source: JMIR Mhealth Uhealth. 2020 Feb 26;8(2):e16266. doi: 10.2196/16266 (PMC7066511; doi:10.2196/16266)
Supplement: Multimedia Appendix 6 [file mhealth_v8i2e16266_app6.docx]

**Multimedia Appendix 6**

Mean changes in efficacy outcomes from baseline in each clinic of the intervention group.

| Outcomes | | | Mean change from baseline^a^, mean (95% CI) | | | | | | | | |
| --- | --- | --- | --- | --- | --- | --- | --- | --- | --- | --- | --- |
|  |  |  | 01  (n=11) | 02  (n=10) | 04  (n=17) | 05  (n=12) | 06  (n=13) | 09  (n=16) | 10  (n=25) | 13  (n=21) | 16  (n=20) |
| Glycemic parameters | | |  |  |  |  |  |  |  |  |  |
|  | HbA_1c_^b^ (%) | | −0.71  (−1.31 to −0.11)^c^ | −0.33  (−1.28 to 0.61) | −0.56  (−0.86 to −0.26)^c^ | −0.76  (−1.07 to −0.45)^c^ | −0.72  (−1.27 to −0.16)^c^ | −0.66  (−1.17 to −0.14)^c^ | −0.80  (−1.08 to −0.51)^c^ | −0.38  (−0.77 to 0.00)^d^ | −0.72  (−1.05 to −0.40)^c^ |
|  | HbA_1c_ (mmol/mol) | | −7.75  (−14.31 to −1.20)^c^ | −3.62  (−13.94 to 6.71) | −6.11  (−9.41 to −2.81)^c^ | −8.29  (−11.69 to −4.89)^c^ | −7.82  (−13.93 to −1.71)^c^ | −7.17  (−12.78 to −1.57)^c^ | −8.71  (−11.80 to −5.62)^c^ | −4.19  (−8.37 to 0.00)^d^ | −7.93  (−11.45 to −4.40) |
|  | FPG^e^ (mg/dL) | | −4.27  (−53.09 to −44.54) | 47.80  (−5.33 to 100.93) | −42.00  (−61.26 to −22.74)^c^ | −21.50  (−41.18 to −1.82)^c^ | −2.92  (−19.52 to 13.68) | −17.00  (−26.39 to −7.61)^c^ | −62.20  (−96.25 to −28.16)^c^ | 5.71  (−45.43 to 34.00) | −13.75  (−28.16 to 0.66) |
| Other metabolic parameters | | |  |  |  |  |  |  |  |  |  |
|  | Weight (kg) | | −0.08  (−1.73 to 1.56) | 1.21  (−1.62 to 4.04) | −1.35  (−2.30 to −0.41)^c^ | −0.01  (−0.84, 0.82) | −1.14  (−2.19 to −0.09)^c^ | −0.86  (−2.07 to 0.36) | −1.70  (−2.35 to −1.05)^c^ | −0.04  (−1.07 to 0.98) | −0.40  (−1.82 to 1.01) |
|  | WC^f^ (cm) | | −0.45  (−2.54 to 1.63) | 0.10  (−0.13 to 0.33) | −2.24  (−3.61 to −0.86)^c^ | −0.58  (−1.03, 2.20) | 0.00  (−1.28 to 1.28) | −2.13  (−5.55 to 1.30) | −2.20  (−3.14 to −1.26)^c^ | −0.90  (−1.79 to −0.02)^c^ | 0.40  (−1.01 to 1.81) |
|  | BMI (kg/m^2^) | | −0.05  (−0.64 to 0.54) | 0.43  (−0.56 to 1.42) | −0.51  (−0.86 to −0.17)^c^ | 0.00  (−0.30, 0.31) | −0.43  (−0.79 to −0.06)^c^ | −0.36  (−0.80 to 0.09) | −0.66  (−0.90 to −0.41)^c^ | −0.01  (−0.37 to 0.35) | −0.21  (−0.75 to 0.33) |
|  | Systolic BP^g^ (mmHg) | | −5.45  (−11.81 to 0.90) | −1.70  (−7.14 to 3.74) | −5.35  (−12.16 to 1.46) | −1.17  (−5.53, 3.20) | −1.62  (−11.42 to 14.65) | 5.94  (−0.37 to 12.25) | −1.20  (−6.54 to 4.14) | 1.43  (−4.39 to 7.25) | 1.85  (−3.24 to 6.94) |
|  | Diastolic BP (mmHg) | | −6.00  (−10.03 to −1.97)^c^ | 0.50  (−4.40 to 5.40) | −3.71  (−7.02 to −0.39)^c^ | −1.67  (−4.77, 1.44) | −2.92  (−10.91 to 5.07) | −1.19  (−6.34 to 3.96) | −2.84  (−6.07 to 0.39) | 1.38  (−3.41 to 6.17) | -2.50  (−7.04 to 2.04) |
|  | Total cholesterol (mg/dL) | | −7.73  (−26.67 to 11.22) | −12.30  (−25.36 to 0.76) | −2.41  (−13.31 to 8.49) | −4.67  (−21.90, 12.57) | −2.08  (−12.00 to 7.85) | −1.13  (−12.10 to 9.85) | −4.68  (−10.51 to 1.15) | −1.33  (−14.73 to 12.07) | 2.55  (−9.10 to 14.20) |
|  | Triglyceride (mg/dL) | | 13.64  (−91.84 to 119.11) | 11.80  (−40.13 to 63.73) | −9.41  (−41.25 to 22.43) | −39.33  (−103.70, 25.03) | −8.08  (−43.63 to 27.48) | −6.44  (−30.80 to 17.92) | −66.24  (−107.74 to −24.74) ^c^ | 4.62  (−42.34 to 51.57) | −14.58  (−35.46 to 6.31) |
|  | HDL^h^ cholesterol (mg/dL) | | 0.64  (−6.03 to 7.30) | −2.00  (−6.78 to 2.78) | −0.76  (−2.54 to 4.07) | 5.17  (−0.93, 11.27) | 3.08  (−4.16 to 10.31) | 0.06  (−3.27 to 3.39) | 4.60  (2.01 to 7.19)^c^ | 3.10  (−0.37 to 6.56) | 3.55  (0.60 to 6.50)^c^ |
|  | LDL^i^ cholesterol (mg/dL) | | −7.55  (−28.50 to 13.41) | −7.10  (17.77 to 3.57) | −1.94  (−11.14 to 7.26) | −3.42  (−17.42, 10.59) | −4.62  (−15.20 to 5.97) | −1.94  (−11.20 to 7.32) | 0.80  (−4.28 to 5.88) | −2.86  (−12.27 to 6.56) | −3.75  (11.93 to 4.43) |
| Questionnaires^j^ | | |  |  |  |  |  |  |  |  |  |
|  | DTSQs^k^ | | 1.73  (−3.34 to 6.79) | 2.60  (−0.14 to 5.34) | 1.59  (−1.64 to 4.82) | 5.50  (0.93,10.07)^c^ | 1.54  (−1.03 to 4.11) | 4.44  (0.62 to 8.26)^c^ | 0.92  (−2.96 to 4.80) | 3.48  (−0.05 to 7.00) | 1.15  (−2.09 to 4.39) |
|  | MMAS-6^l^ | | 0.00  (−0.74 to 0.74) | 0.40  (−0.68 to 1.48) | 0.71  (0.06 to 1.36)^c^ | 0.58  (−0.52, 1.68) | 0.46  (−0.07 to 0.99) | 0.94  (0.44 to 1.43)^c^ | 0.68  (0.15 to 1.21)^c^ | 0.19  (−0.34 to 0.72) | 0.55  (−0.25 to 1.35) |
|  |  | Motivation | 0.36  (−0.09 to 0.82) | 0.00  (−0.95 to 0.95) | 0.35  (−0.19 to 0.90) | 0.50  (−0.24, 1.24) | 0.08  (−0.44 to 0.60) | 0.44  (0.16 to 0.71)^c^ | 0.56  (0.20 to 0.91)^c^ | 0.19  (−0.26 to 0.64) | 0.70  (0.24 to 1.16)^c^ |
|  |  | Knowledge | -0.36  (−0.91 to 0.18) | 0.40  (−0.10 to 0.90) | 0.35  (0.04 to 0.66)^c^ | 0.08  (−0.55, 0.72) | 0.38  (−0.25 to 1.02) | 0.50  (0.06 to 0.94)^c^ | 0.12  (−0.18 to 0.42) | 0.00  (−0.20 to 0.20) | −0.15  (−0.70 to 0.40) |

^a^Assessed using the paired *t* test

^b^HbA_1c_: hemoglobin A_1c_.

^c^*P* <.05.

^d^*P* =.05.

^e^FPG: Fasting plasma glucose.

^f^WC: waist circumference.

^g^BP: blood pressure.

^h^HDL: high-density lipoprotein.

^i^LDL: low-density lipoprotein

^j^Higher DTSQs and MMAS-6 scores indicate a favorable state.

^k^DTSQs: Diabetes Treatment Satisfaction Questionnaire status version.

^l^MMAS-6: 6-item Morisky Medication Adherence Scale.
