# Supplementary material for: Effect of a Mobile Phone–Based Glucose-Monitoring and Feedback System for Type 2 Diabetes Management in Multiple Primary Care Clinic Settings: Cluster Randomized Controlled Trial
Source: JMIR Mhealth Uhealth. 2020 Feb 26;8(2):e16266. doi: 10.2196/16266 (PMC7066511; doi:10.2196/16266)

**Multimedia Appendix 7**

Subgroup analyses of changes in fasting plasma glucose by sex, age, BMI, and baseline hemoglobin A_1c_.
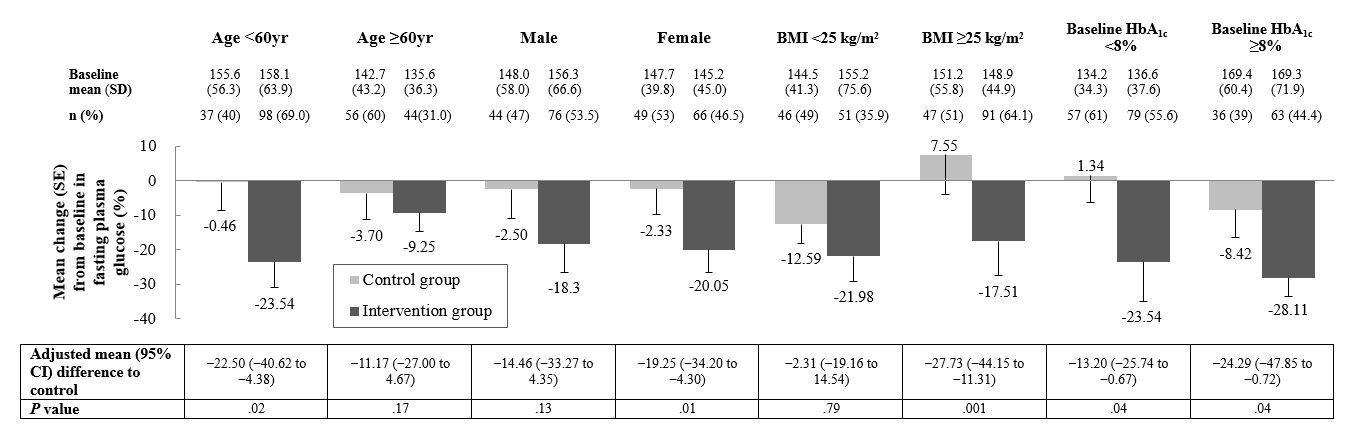

Supplement: Multimedia Appendix 7 [file mhealth_v8i2e16266_app7.docx]
